# Early-phase administration of human amnion-derived stem cells ameliorates neurobehavioral deficits of intracerebral hemorrhage by suppressing local inflammation and apoptosis

**DOI:** 10.1186/s12974-022-02411-3

**Published:** 2022-02-12

**Authors:** Yoji Kuramoto, Mitsugu Fujita, Toshinori Takagi, Yuki Takeda, Nobutaka Doe, Kenichi Yamahara, Shinichi Yoshimura

**Affiliations:** 1 Department of Neurosurgery, Hyogo College of Medicine, 1–1 Mukogawa-cho, Nishinomiya, Hyogo 663–8501 Japan; 2 Center for Medical Education and Clinical Training, Kindai University Faculty of Medicine, 377-2 Oonohigashi, Osakasayama, Osaka 589–8511 Japan; 3 Laboratory of Neurogenesis and CNS Repair, Hyogo College of Medicine, 1-1 Mukogawa-cho, Nishinomiya, Hyogo 663–8501 Japan; 4 Laboratory of Psychology, General Education Center, Hyogo University of Health Science, 1-3-6 Minatojima, Chuo-ku, Kobe, Hyogo 650–8530 Japan; 5 Laboratory of Medical Innovation, Institute for Advanced Medical Sciences, Hyogo College of Medicine, 1-1 Mukogawa-cho, Nishinomiya, Hyogo 663–8501 Japan

**Keywords:** Human amnion-derived stem cell, Intracerebral hemorrhage, Macrophage, Microglia, Apoptosis, Inflammation

## Abstract

**Background:**

Intracerebral hemorrhage (ICH) is a significant cause of death and disabilities. Recently, cell therapies using mesenchymal stem cells have been shown to improve ICH-induced neurobehavioral deficits. Based on these findings, we designed this study to evaluate the therapeutic efficacy and underlying mechanisms by which human amnion-derived stem cells (hAMSCs) would ameliorate neurobehavioral deficits of ICH-bearing hosts.

**Methods:**

hAMSCs were induced from amnia obtained by cesarean section and administered intravenously to ICH-bearing mice during the acute phase. The mice were then subject to multitask neurobehavioral tests at the subacute phase. We attempted to optimize the dosage and timing of the hAMSC administrations. In parallel with the hAMSCs, a tenfold higher dose of human adipose-derived stem cells (ADSCs) were used as an experimental control. Specimens were obtained from the ICH lesions to conduct immunostaining, flow cytometry, and Western blotting to elucidate the underlying mechanisms of the hAMSC treatment.

**Results:**

The intravenous administration of hAMSCs to the ICH-bearing mice effectively improved their neurobehavioral deficits, particularly when the treatment was initiated at Day 1 after the ICH induction. Of note, the hAMSCs promoted clinical efficacy equivalent to or better than that of hADSCs at 1/10 the cell number. The systemically administered hAMSCs were found in the ICH lesions along with the local accumulation of macrophages/microglia. In detail, the hAMSC treatment decreased the number of CD11b^+^CD45^+^ and Ly6G^+^ cells in the ICH lesions, while splenocytes were not affected. Moreover, the hAMSC treatment decreased the number of apoptotic cells in the ICH lesions. These results were associated with suppression of the protein expression levels of macrophage-related factors iNOS and TNFα.

**Conclusions:**

Intravenous hAMSC administration during the acute phase would improve ICH-induced neurobehavioral disorders. The underlying mechanism was suggested to be the suppression of subacute inflammation and apoptosis by suppressing macrophage/microglia cell numbers and macrophage functions (such as TNFα and iNOS). From a clinical point of view, hAMSC-based treatment may be a novel strategy for the treatment of ICH.

**Supplementary Information:**

The online version contains supplementary material available at 10.1186/s12974-022-02411-3.

## Background

Intracerebral hemorrhage (ICH) accounts for 10–20% of all strokes [1, 2]; its mortality rate after 1 month of onset is about 40% and its disability rate after 3 months of onset is about 60% [3, 4]. Although surgical interventions and acute medications are effective as life-saving care, they are less effective in improving neurobehavioral symptoms [5]. Rehabilitation has been a priority for functional recovery of the ICH patients, but its therapeutic effectiveness is limited [6].

In recent years, various therapies using stem cells have been investigated and developed. Stem cells have various functions, including proliferation and self-renewal under certain conditions [7]. Stem cells are subcategorized into two major groups, depending on their stage of development. One is the embryonic stem cells that can be isolated from the inner cell mass of the blastocyst [7]. The another is the somatic stem cells, which originates from various adult tissues. Among the various types of somatic stem cells, mesenchymal stem cells (MSCs) exert unique biological effects such as the production of growth factors and cytokines, immunomodulation, neurogenesis, and angiogenesis [8]. Since MSCs are present in various tissues, such as bone marrow, adipose, and perinatal tissues, their application to various diseases is one of the major research targets. Human bone marrow-derived stem cells (hBMSCs) are representative MSCs that are approved for treating graft-versus-host disease (GVHD) in many countries. Clinical trials are being conducted for various diseases including brain stroke [9]. Several animal studies have already shown that intravenous administration of MSCs improves neurobehavioral functions after ICH [10, 11].

The disadvantage of hBMSCs is that they require a clinically invasive bone marrow aspiration, with 0.05% complications such as bleeding and infection associated with the procedure [12]. To solve this problem, we have previously established human adipose-derived stem cells (hADSCs) that is less invasively harvestable with comparable or superior abilities to produce cytokines to hBMSCs [13]. Thereafter, we have established human amnion-derived stem cells (hAMSCs) that are isolated from fetal appendages that are often discarded after a cesarean section [14]. hAMSC isolation do not require extra invasive procedures. In addition, hAMSCs have been found to secrete a variety of cytokines at higher levels comparable to hBMSCs [15] and hADSCs [16]. The immunomodulatory effects of hAMSCs on T_H_1/T_H_17 immune responses are also comparable to those of hBMSCs [17, 18]. Preclinical in vivo studies revealed no significant adverse events associated with the hAMSC administration [19, 20]. No major problems have been reported in human safety study [21]. Indeed, clinical trials of hAMSC are being conducted for GVHD and Crohn’s disease [19, 22].

The above findings led us to hypothesize that intravenously administration of hAMSCs would improve the ICH-induced neurobehavioral deficits by altering local inflammation. To address this hypothesis, we induced ICH in the mouse brain, treated the mice with hAMSCs, and subjected the mice to multitask neurobehavioral tests. To clarify the underlying mechanisms of hAMSC-based treatment, we conducted immunostaining, TUNEL staining, flow cytometry, and Western blotting.

## Methods

### Cell preparation of hAMSCs and hADSCs

The Ethics Committee of Hyogo College of Medicine approved this study (approval numbers: 325 and 1880). The procedures of hAMSCs and hADSCs have been described previously [11, 14]. Briefly, we first obtained written informed consent from the donors; for hAMSC, pregnant women waiting for cesarean section, and for hADSCs, patients who underwent abdominal surgeries. Regarding hAMSCs, human fetal membranes were obtained by cesarean section. Amnia were detached mechanically from the chorion and digested with collagenase/dispase solution for 1 h at 37 °C in a water bath shaker. The cells were filtered through a 100-μm mesh filter, resuspended in α-minimal essential medium (α-MEM; Invitrogen, CA) supplemented with 10% bovine-derived platelet lysate “NeoSERA” (Japan Biomedical Co., Ltd, Japan), plated on dishes, and incubated at 37 °C with 5% CO_2_. Spindle-shaped cells formed visible colonies in 1–2 days. Regarding hADSCs, adipose tissues were obtained during abdominal surgeries, rinsed, cut, homogenized, digested with liberase. Stromal vascular fraction was obtained, the cells were stocked at − 80 °C with STEM-CELLBANKER (Takara Bio) after the fourth passage.

### Mice and ICH induction

The Institutional Animal Care Ethical Committee approved all the animal experiments of this study (Approval numbers: 17-034 and 19-048). The procedure has been described previously [23]. Briefly, 7–9-week-old male C57BL/6J mice were housed under photocyclic conditions for 12 h and fed with freely accessible water and food (CLEA Japan, Inc, Tokyo, Japan). The mice were anesthetized with 1.5–2.0% isoflurane, and ICH was induced by injecting 0.4 units of collagenase AOF type A (Worthington) into the brains of mice located 2 mm to the left and 3.5 mm depth from the bregma.

### Administration of hAMSC or hADSC to ICH-bearing mice

We conducted two experiments to optimize the dosage and timing of the hAMSC administrations. To optimize the dosage of hAMSCs and compare them with the previously studied hADSCs, mice were randomly divided into five groups as follows: the high-dose hAMSC group (*n* = 24), the low-dose hAMSC group (*n* = 11), the hADSC group (*n* = 11), the untreated ICH group (*n* = 32), and sham control group (*n* = 32). Here, the high-dose hAMSC group and low-dose group received intravenous injections of hAMSC at doses of 1.0 × 10^5^ cells/50 μl and 2.5 × 10^4^ cells/50 μl, respectively, at 24 h after the ICH induction. The hADSC group received an intravenous injection of hADSC at doses of 1.0 × 10^6^ cells/100 μl at 24 h after the ICH induction according to previous study [11]. The untreated ICH group received an intravenous injection of 50 μl of vehicle control. The sham group underwent a scalp incision and trepanation alone.

The second experiment was designed to optimize the timing of the hAMSC administration. Mice were randomly divided into four groups: the D1-hAMSC group (*n* = 12), the D3-hAMSC group (*n* = 12), the untreated ICH group (*n* = 11), and sham control group (*n* = 12). Here, the D1-hAMSC and D3-hAMSC group received injected intravenous injections of hAMSCs at doses of 1.0 × 10^5^ cells/50 μl at 24 and 72 h, respectively, after the ICH induction. The untreated ICH group received an intravenous injection of 50 μl of vehicle control. The sham control group underwent a scalp incision and trepanation alone.

In some experiments, mice received intravenous administration of TNFα (1 µg/10 µl) simultaneously with hAMSCs (1.0 × 10^5^ cells/50 µl) and hAMSCs at 24 h after the ICH induction. After 72 h of intravenous administration, the mice were killed and TUNEL staining was conducted as described below.

### Neurobehavioral tests

The procedure has been described previously [11, 24]. Briefly, the following neurobehavioral tests were started at Day 29 to assess the phenotypic differences in the mice. Researchers who were blinded to the experiments evaluated the results of the tests. Details of each test are described below.

### Open space swimming test

The open space swimming test was conducted to evaluate their neuromuscular strength and depression-like symptoms of the mice. A circular pool (inside diameter: 95 cm, depth: 35 cm) surrounded by a white wall (width: 130 cm, height: 120 cm) was filled with water to a depth of 20 cm. The water was made opaque with titanium oxide to enable a video-tracking system (Be Chase ver.1.3, ISONIX) to trace the behavior of mice. The temperature of the water was maintained at 22 ± 1 °C. In each test, a mouse was placed in the pool with its head facing the outer edge of the pool and allowed to swim freely for 10 min. All tests were recorded with an overhead CCD camera. The total swimming length of the subjected mouse was calculated using the video-tracking system.

### Morris water maze learning test

The Morris water maze learning test was conducted to evaluate the spatial recognition of the mice. A circular pool (inside diameter: 95 cm, depth: 35 cm) surrounded by a white wall (width: 150 cm, height: 120 cm) was filled with water to a depth of 22 cm. The water was made opaque with titanium oxide to enable the video-tracking system to trace the behavior of mice. The temperature of the water was maintained at 24 ± 1 °C. The pool was divided into four virtual quadrants: north, south, east, and west. A round white platform (diameter: 10 cm) was placed at the center of the north quadrant of the pool and submerged 0.5 cm below the surface of the water. In each test, a mouse was randomly released into the water with its head facing the edge of the pool in either the south, east, or west quadrants. The test ended when the subjected mouse reached the platform and stayed on it for 10 s. If the mouse could not find the platform within 60 s, it was guided to the platform by the experimenter and kept there for 10 s. Each mouse was tested five times per day with 30-s intervals for 5 consecutive days. All tests were recorded with an overhead CCD camera. The duration between the release of the subjected mouse into the water and its arrival on the platform was measured as the escape latency using the video-tracking system.

### Passive avoidance learning test

The passive avoidance learning test was conducted to evaluate the long-term memory function of the mice. An apparatus consisted of light and dark compartments with the same dimension (15 × 15 × 15 cm) with a grid floor. A guillotine door separated the two compartments. In the conditioning test, a mouse was placed in the light compartment. Ten sec later, the guillotine door was opened. When the mouse moved into the dark compartment, the guillotine door was closed. Ten sec later, a scrambled electrical shock (at 120 V for 5 s) was delivered through the grid floor. Twenty-four and 48 h later, a retention test was conducted without the electrical shock. The subjected mouse was placed in the light compartment and the duration to enter the dark compartment was recorded up to 180 s. This duration (passive avoidance latency) reflected the memory functions of the subjected mice to electrical shocks with 24-h intervals.

### Immunostaining

The procedure has been described previously [11]. Mice were divided into two groups: the hAMSC group that received an intravenous injection of 1.0 × 10^5^ hAMSCs in 50 μl of vehicle control at 24 h after the ICH induction, and the ICH group that received an intravenous injection of 50 μl of vehicle control alone. The mice were killed at Days 4 and 8 after the ICH inductions. The mice were perfused with normal saline and then 4% paraformaldehyde in PBS under deep anesthesia with 3–4% isoflurane. Brain tissues were fixed for 1 day with 4% paraformaldehyde after removal and then transferred to 30% sucrose solution at 4 °C. The brain tissue was sectioned in 8-μm thickness using a cryostat (CM1950, Leica Biosystems). The primary antibodies used in this experiment were as follows: anti-Iba1 (ab178847, abcam) at 1:250 dilution and anti-human KU80 (STEM101, Takara Bio) at 1:100 dilution. The second antibodies used were as follows: Alexa 488-labeled anti-rabbit (CST) and Alexa 555-labeled anti-mouse (CST). DAB staining was conducted using DAB peroxidase substrate kit (SK-4100, Vector). The immunostaining assay was visualized using a fluorescence microscope (BZ-x710, Keyence).

### TUNEL staining

The procedure has been described previously [25]. Briefly, frozen brain sections were immobilized with 3% H_2_O_2_/100 μl methanol. Then, the sections were incubated for 1 h at room temperature with TUNEL reaction mixture that includes terminal deoxynucleotidyl transferase (TdT) and fluorescein-conjugated dUTP (In Situ Cell Death Detection Kit POD; Sigma-Aldrich). The sections were washed with PBS and incubated for 30 min at room temperature with anti-fluorescein antibodies conjugated with peroxidase. Then, peroxidase reacted by DAB peroxidase substrate KIT, and the images were visualized using a microscope (BZ-x710, Keyence).

### Flow cytometry

The procedure has been described previously [11]. Briefly, mice were perfused with PBS under deep anesthesia with 3–4% isoflurane, and the brains and spleens were extracted. The brains were cut into small pieces and passed by 18-gauge and 20-gauge needles. The minced brain tissue was washed, resuspended in 25% Percoll (GE Healthcare), and centrifuged for 20 min at 500×*g*. The supernatants were discarded. The cell pellets were resuspended in Histopaque (Sigma) and centrifuged for 20 min at 500×*g*. Spleens were minced, filtered by 70-μm mesh filters, resuspended in Histopaque, and centrifuged for 20 min at 500×*g*. For both, enriched immune cells were recovered at the Histopaque interface. The cells were corrected and incubated with the following antibodies: PerCP-Cy5.5-conjugated anti-mouse CD11b monoclonal antibody (BD Pharmingen), phycoerythrin (PE)-Cy7-conjugated anti-mouse CD45 monoclonal antibody (BD Pharmingen), APC-conjugated anti-mouse Ly-6C monoclonal antibody (BD Pharmingen), and BV421-conjugated anti-mouse Ly-6G monoclonal antibody (BD Horizon). Isotype controls were also used. The fluorescent-labeled cells were analyzed by LSRFortessaX-20 (BD Biosciences) and BD FACSDiva software (BD Biosciences). Analysis of flow cytometry results was conducted on FlowJo software version 10.5 (BD Bioscience).

### Protein isolation and Western blotting

The procedure has been described previously [26, 27]. Briefly, mice were divided into two groups as described above: the hAMSC group and the ICH group. The mice were killed with deep anesthesia with 3–4% isoflurane, and brain tissues were extracted and frozen immediately in liquid nitrogen. Proteins were isolated from intracerebral hematoma and perihematomal brain tissue using RIPA buffer (ATTO, WSE-7420) and denatured at 95 °C for 5 min. Next, denatured proteins were electrophoresed and transferred to PVDF membranes. Membrane blocking was conducted using EzBlock Chemi (ATTO, AE-1475) at room temperature for 30 min. The primary antibodies used in this study were as follows: inducible nitric oxide synthase: arginase1(CST, 93668) at 1:1000, iNOS (CST, 13120) at 1:1000, TNFα (Santa Cruz, SC-52746) at 1:1000, cleaved caspase 3 (Santa Cruz, SC-56053) at 1:200 dilution, Phospho-Akt (pAkt; CST, 4060) at 1:2000 dilution, p38 MARK (CST, 9212) at 1:1000 dilution, pNFκβ (CST, 3039) at 1:1000 dilution, NFκβ (CST, 8242) at 1:1000 dilution, pSTAT3 [Tyr705] (CST, 9145) at 1:1000 dilution, pSTAT3 [Ser727] (CST,9134) at 1:1000 dilution, STAT3 (CST, 30835) at 1:1000 dilution, pAkt XP (CST,4060) at 1:1000 dilution, Akt (CST, 4685) at 1:1000 dilution and β-actin (Sigma, A5441) at 1:5000 dilution. The secondary antibodies used were as follows: HRP-conjugated goat anti-rabbit IgG (H+L) (Thermo Scientific, 32460) at 1:1000 dilution and HRP-conjugated goat anti-mouse IgG (H+L) (Thermo Scientific, 32430) at 1:500 dilution. HRP luminescence was detected by Ez WestLumi plus (ATTO, WSE-7120L) and Luminograph1 (ATTO, WSE-6100). The protein expression levels of the ICH group on Day 4 were used as control.

### Statistical analysis

All the results were expressed as mean ± SEM. For the statistical analysis of the neurobehavioral tests, repeated measures ANOVA was conducted for groups as the between-subject factor and repeated measures (e.g., session, trial, or time) as the within-subject factor. When ANOVA found significant effects, post hoc comparison test was conducted using Tukey–Kramer method. For the statistical analyses of flow cytometry data and immunostaining date, we conducted the Wilcoxon test on each Days 2, 4, and 8. For the statistical analyses of Western blotting, we conducted Tukey–Kramer test. Significance level was set at *P* < 0.05 (two-tailed). These statistical analyses were conducted on JMP version 15 (SAS Institute).

## Results

### Intravenous administration of hAMSCs dose-dependently improves ICH-induced neurobehavioral deficits during the subacute phase

**Fig. 1:**
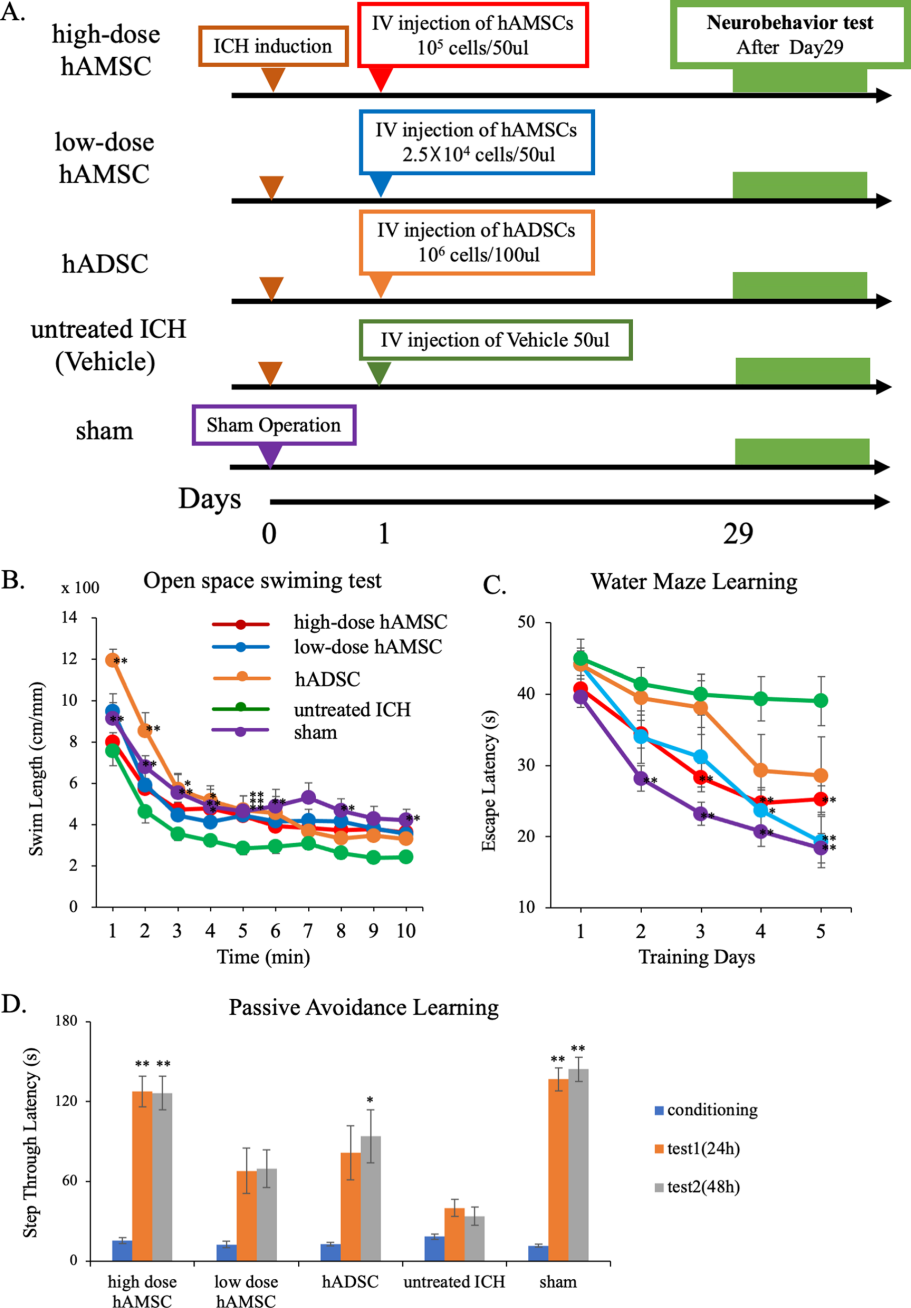
Intravenous administration of hAMSCs dose-dependently improves ICH-induced neurobehavioral deficits during the subacute phase. **A** A protocol of neurobehavioral tests to optimize the administration dose of hAMSCs for mice bearing intracranial hemorrhage (ICH). The mice were divided into five groups: high-dose hAMSC group (*n* = 24), low-dose hAMSC group (*n* = 11), hADSC group (*n* = 11), ICH group (*n* = 32), and sham group (*n* = 32). The mice were then subject to the following neurobehavioral tests at Day 29 and later: open space swimming test (**B**), water maze learning test (**C**), and passive avoidance learning test (**D**). Data are plotted in mean ± SEM. *P* values are based on Tukey–Kramer test. *P < 0.05 and **P < 0.01 compared with the ICH group

To evaluate the therapeutic efficacy and optimal dosage of hAMSCs for ICH, we induced ICH in the mouse brain and treated the mice with intravenous administration of various doses of hAMSCs. Based on the previous study where hAMSCs have been administered at doses of 1.0 × 10^6^ and 4.0 × 10^6^ cells per kg of body [19, 22], the administration doses of hAMSCs used in this study were calculated and determined as 2.5 × 10^4^ and 1.0 × 10^5^ per mouse. In contrast, hADSCs have been used to ICH-bearing mice at a dose of 1.0 × 10^6^ hADSCs per mouse [11]. That is, we administered hAMSCs at 2.5 × 10^4^ (1/40) and 1.0 × 10^5^ (1/10) per mouse compared with the dose of hADSCs (1.0 × 10^6^ per mouse). Subsequently, to evaluate neurobehavioral symptoms, the following multitasking behavioral tests were conducted during subacute phase: open space swimming test, water maze learning test, and passive avoidance learning test. The open space swimming test suggested significant effects of the group on swimming length (Fig. 1B; *F*_4, 106_ = 9.5227, *P* = 0.0022) and swimming time (*F*_9, 98_ = 46.6700, *P* < 0.0001). The Morris water maze learning test suggested significant effects of the group (Fig. 1C; *F*_4, 94_ = 9.3334, *P* < 0.0001) and the latency (*F*_4, 91_ = 30.3615, *P* < 0.0001), with the findings that the high-dose hAMSC group showed shorter latency than the untreated ICH group at Day 3 (*P* = 0.0073), Day 4 (*P* = 0.0048), and Day 5 (*P* = 0.0092) and that the low-dose hAMSC group showed shorter latency at Day 4 (*P* = 0.0208) and Day 5 (*P* = 0.0016). The passive avoidance learning test suggested significant effects of group (Fig. 1D; *F*_4, 94_ = 20.8277, *P* < 0.0001), with the findings that the high-dose hAMSC group showed longer latency than the untreated ICH group at 24 h (*P* < 0.0001) as well as 48 h (*P* < 0.0001) and that the hADSC also showed longer latency than the untreated ICH group on 48 h (*P* = 0.0186). These data suggest that the hAMSCs administration would promote comparable efficacy to the hADSCs administration at 1/10 to 1/40 of the cell number.

### Early intravenous administration of hAMSCs might improve ICH-induced neurobehavioral deficits during the subacute phase

**Fig. 2:**
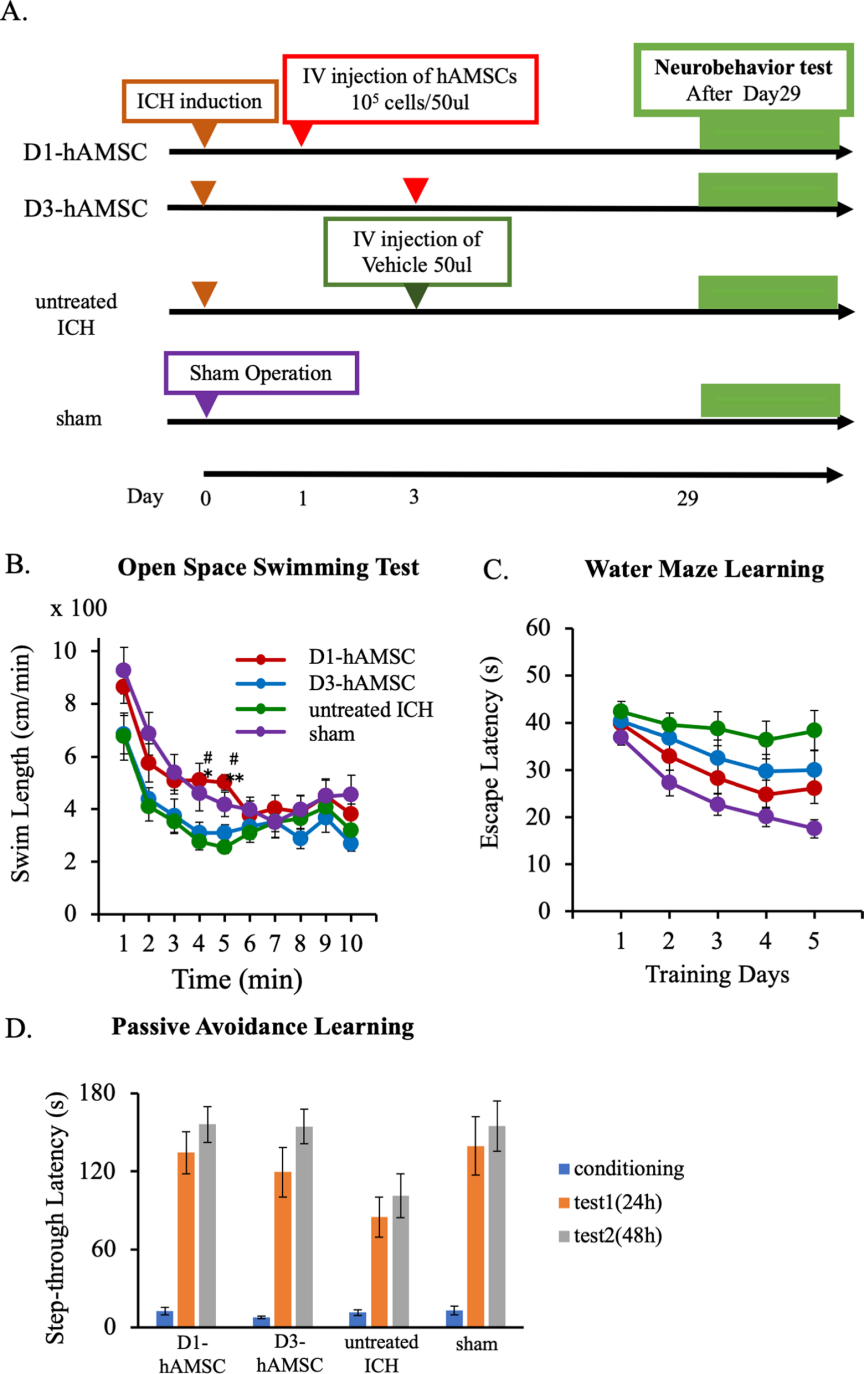
Early intravenous administration of hAMSCs might improve ICH-induced neurobehavioral deficits during the subacute phase. **A** A protocol of neurobehavioral tests to optimize the timing of the hAMSC administration. The ICH-bearing mice were divided into four groups: D1-hAMSC group (*n* = 12), D3-hAMSC groups (*n* = 12), untreated ICH group (*n* = 11), and sham group (*n* = 12). The mice were then subject to the following neurobehavioral tests at Day 29 and later: open space swimming test (**B**), water maze learning test (**C**), and passive avoidance learning test (**D**). Data are plotted in mean ± SEM. *P* values are based on Tukey–Kramer test. *P < 0.05 and **P < 0.01 compared with the ICH group. ^#^*P* < 0.05 and ^##^*P* < 0.01 compared with the D3-hAMSC group

Next, we aimed at optimizing the timing of hAMSC treatment. To this end, we treated with intravenously hAMSC administration of two timings (Days 1 and 3 after the ICH induction), and the ICH-bearing mice were subjected to the same multitask neurobehavioral test described above. The open space swimming test suggested significant effects of the group on swimming lengths (Fig. 2B; *F*_3, 44_ = 3.966, *P* = 0.0138) and time (*F*_9, 36_ = 15.724, *P* < 0.0001), with the findings that the D1-hAMSC group swam longer than the untreated ICH group (*P* = 0.0145) as well as the D3-hAMSC group (*P* = 0.0452) at 4 min. The Morris water maze learning test suggested significant effects of the group (Fig. 2C; *F *_3, 44_ = 6.496, *P* = 0.0001), with the findings that the D1-hAMSC group tended to have shorter latency than the untreated ICH group at Day 5 (*P* = 0.0872). The passive avoidance-learning test suggested significant effects of the group (Fig. 2D; *F*_3, 44_ = 4.7152, *P* = 0.00061), with the findings that the D1-hAMSC and the D3-hAMSC groups tended to have longer latency than the untreated ICH group at 48 h (*P* = 0.0526 and *P* = 0.063, respectively). Taken together, these data suggest initiating the hAMSC treatment in Day 1 led to better therapeutic outcome compared with Day 3.

### A small number of hAMSCs pass through the blood–brain barrier and interact with macrophage or microglial cell

**Fig. 3:**
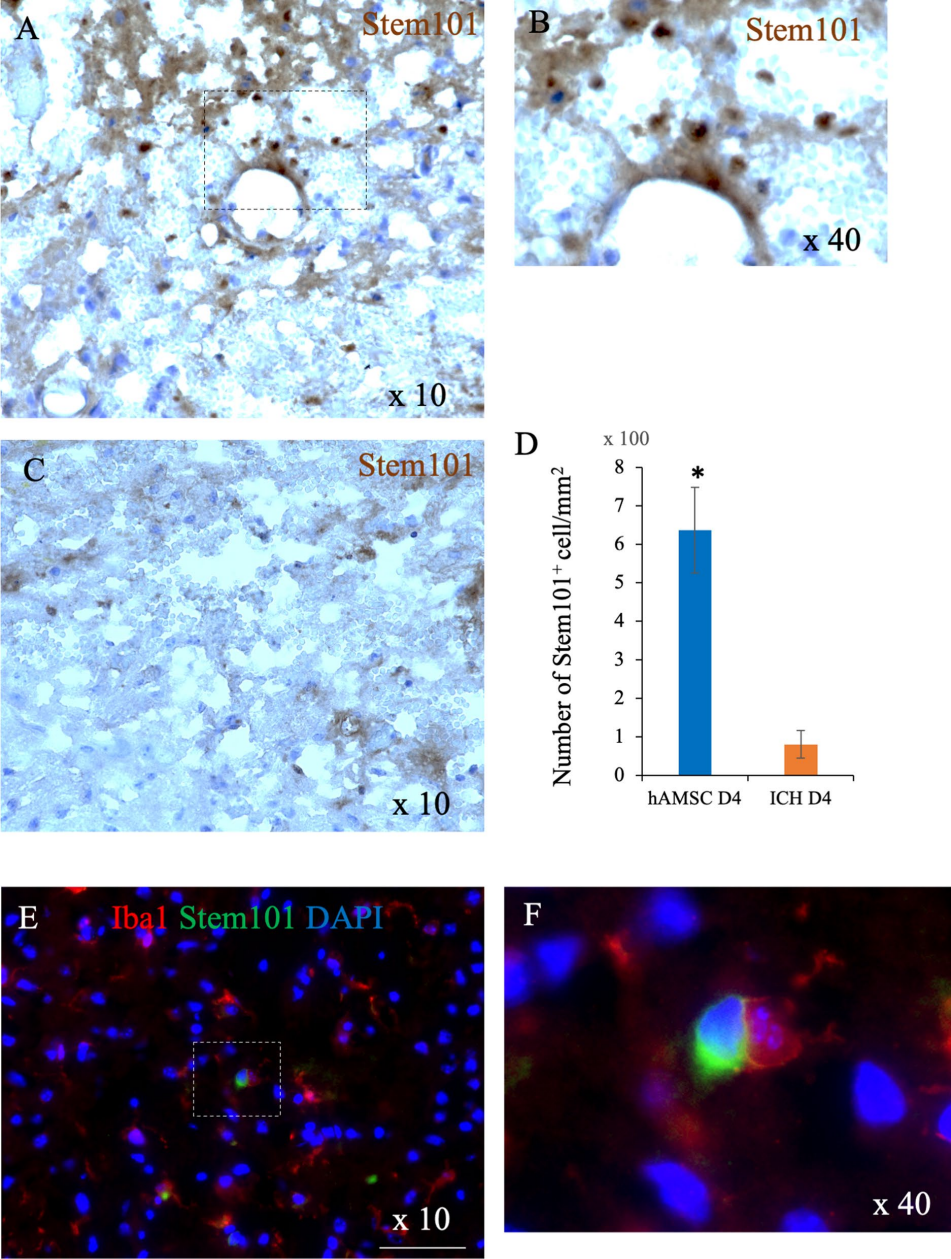
A small number of hAMSCs pass through the blood–brain barrier and interact with macrophage or microglial cell. Representative images of Stem101 staining of the ICH lesions at Day 4. **A** The hAMSC group, magnification: ×10. **B** The hAMSC group, magnification: ×40. **C** The ICH group, magnification: ×10. **D** The number of STEM101-reactive cells around the ICH lesions were enumerated. Double staining of Stem101 and Iba1 in the ICH lesion in the hAMSC group (**E**, **F**). Scale bars indicate 100 μm

To clarify that hAMSCs enter the brain directly on the ICH, we attempted to visualize the presence of hAMSCs in the brain by anti-human Ku80 (STEM101) immunostaining. Here, STEM101 is an antibody that reacts specifically with nucleic molecule Ku80 in human cells but not cross-react with murine cells. Intravenous administration of hAMSCs accumulate STEM101-reactive cells around the vascular structure at Day 4 (Fig. 3A, B). This finding was not observed in the untreated ICH group (Fig. 3C). The hAMSC group showed STEM101-reactive cells at significantly higher levels than the control group (*P* = 0.0495, Fig. 3D). In addition, double staining with Stem101 and anti-Iba1 antibody showed the co-existence of hAMSCs with macrophages/microglia in the ICH lesion (Fig. 3E, F). These findings suggested that hAMSCs would migrate to the ICH-lesion of the brain to interact with macrophages/microglia.

### hAMSC administration decreases the number of CD11b^+^CD45^+^ cells, Ly6C^+^ and Ly6G^+^ cells in the ICH lesions

**Fig. 4:**
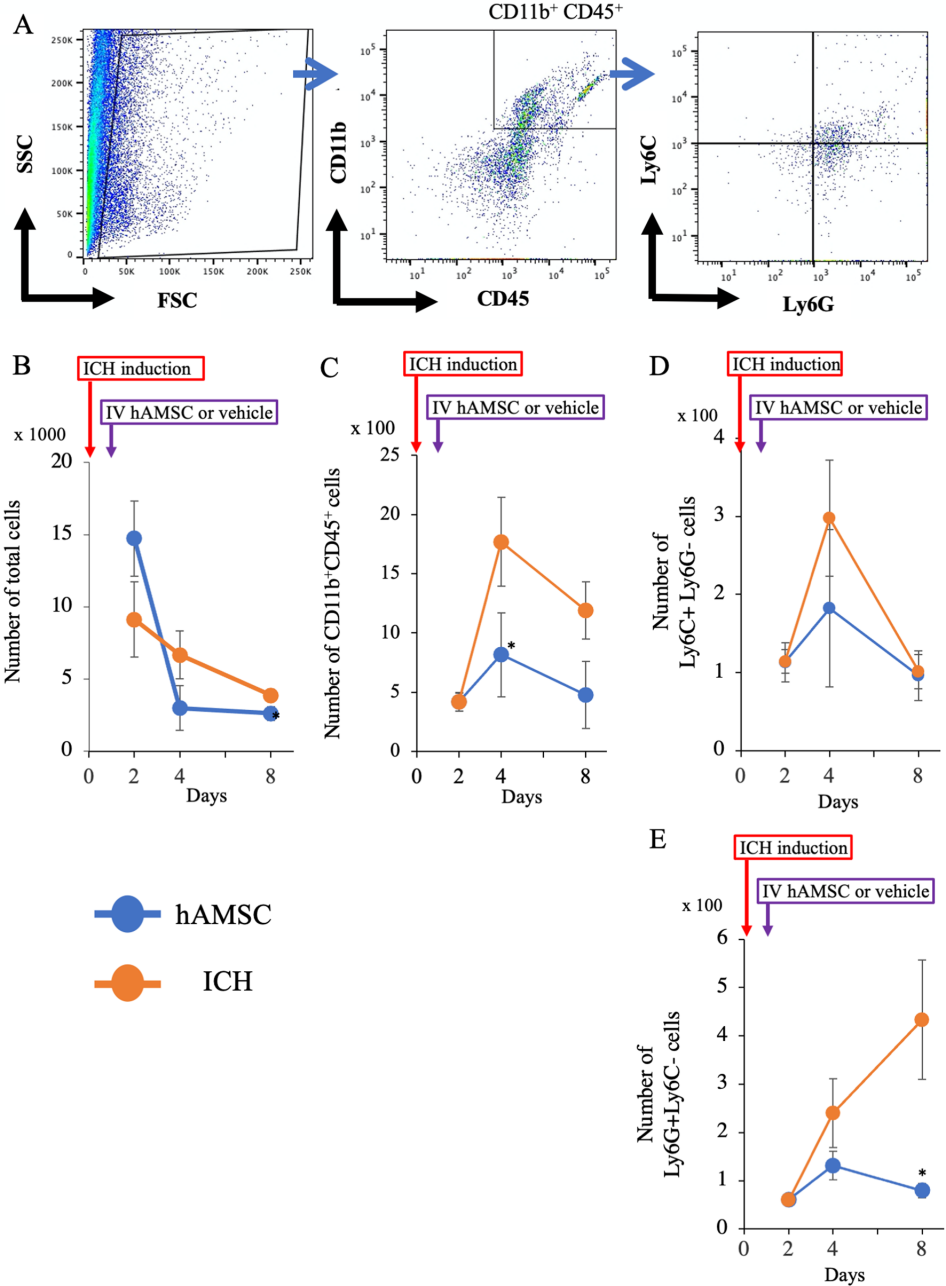
A hAMSC administration decreases the number of CD11b^+^CD45^+^ cells, Ly6C^+^ and Ly6G^+^ cells in the ICH lesions. **A** Representative gating strategy of the flow cytometry for CD11b CD45 Ly6C Ly6G cells. Data were obtained from the ICH lesions at Days 2, 4 and, 8. **B** The total cell number. **C** The number of CD11b^+^CD45^+^ cells. **D** The number of CD11b^+^CD45^+^Ly6C^+^Ly6G^−^ cells. **E** The number of CD11b^+^CD45^+^Ly6C^−^Ly6G^+^ cells. Data are plotted in mean ± SEM. *P* values are based on the Wilcoxon test. *P < 0.05 compared with the ICH group

The above findings led us to hypothesize that the hAMSCs would improve the neurobehavioral deficits of ICH-bearing mice by altering local inflammation. To address this hypothesis, we performed flow cytometry for CD11b^+^ cells that include macrophages, microglia, and monocytes [28]. The hAMSC administration decreased total cell number at Day 8 (*P* = 0.0475, Fig. 4B) and CD11b^+^CD45^+^ cells at Day 4 (*P* = 0.0433, Fig. 4C). We further examined the subpopulations of CD11b^+^CD45^+^ cells for their polarization. The hAMSC administration tended to decrease the number of Ly6C^+^ Ly6G^−^ cells at Day 4 (*P* = 0.075, Fig. 4D) and significantly decreased the number of Ly6G^+^ Ly6C^−^ cells at Day 8 (*P* = 0.012, Fig. 4E) compared with the ICH group. These data suggest that the hAMSC administration would decrease the number of CD11b^+^CD45^+^ cells as well as Ly6C^+^ and Ly6G^+^ cells in the ICH lesion.

### hAMSC administration does not affect CD11b^+^CD45^+^ cells in the spleen

**Fig. 5:**
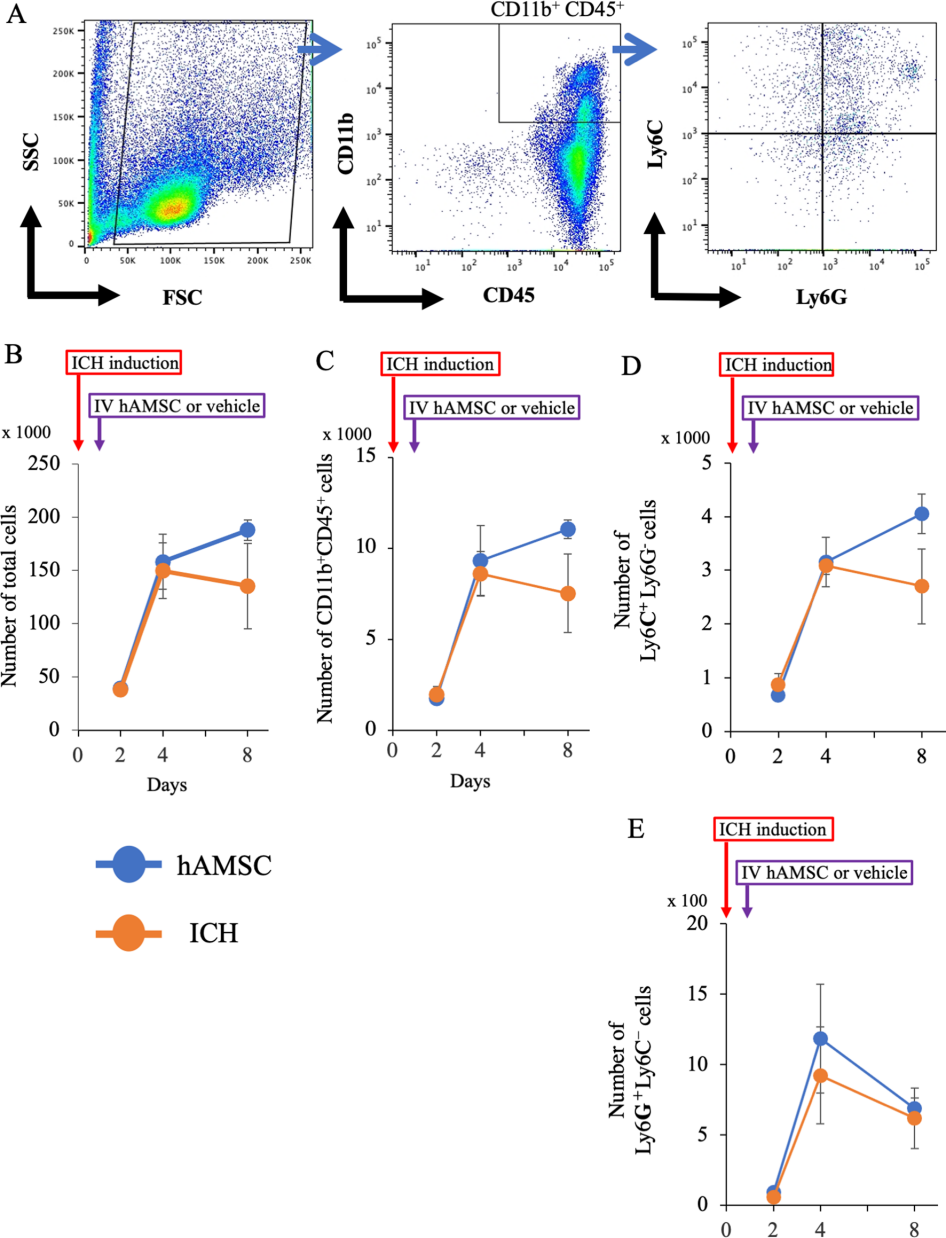
hAMSC administration does not affect CD11b^+^CD45^+^ cells in the spleen. **A** Representative gating strategy for the flow cytometry of CD11b CD45 Ly6C Ly6G cells. Data were obtained from spleen at Days 2, 4 and, 8. **B** The total cell number. **C** The number of CD11b^+^CD45^+^ cells. **D** The number of CD11b^+^CD45^+^Ly6C^+^Ly6G^−^ cells. **E** The number of CD11b^+^CD45^+^Ly6C^−^Ly6G^+^ cells. Data are plotted in mean ± SEM. *P* values are based on the Wilcoxon test. *P < 0.05 compared with the ICH group

To evaluate the systematic immune responses, we also analyzed splenic CD11^+^CD45^+^ cells in the hAMSC group and the ICH group by flow cytometry (Fig. 5). Both the groups exhibited no differences in the total cell number (Fig. 5B) and CD11b^+^CD45^+^ cells (Fig. 5C). We also evaluated the number of Ly6C^+^Ly6G^−^, and Ly6G^+^Ly6C^−^ cells (Fig. 5D, E) in the CD11b^+^CD45^+^ cells and observed no significance. These data suggest that the hAMSC administration did not affect systemic immune responses and that ICH-induced inflammation was localized in the brain.

### hAMSCs administration decreases the TUNEL-positive cells in the ICH lesions

**Fig. 6:**
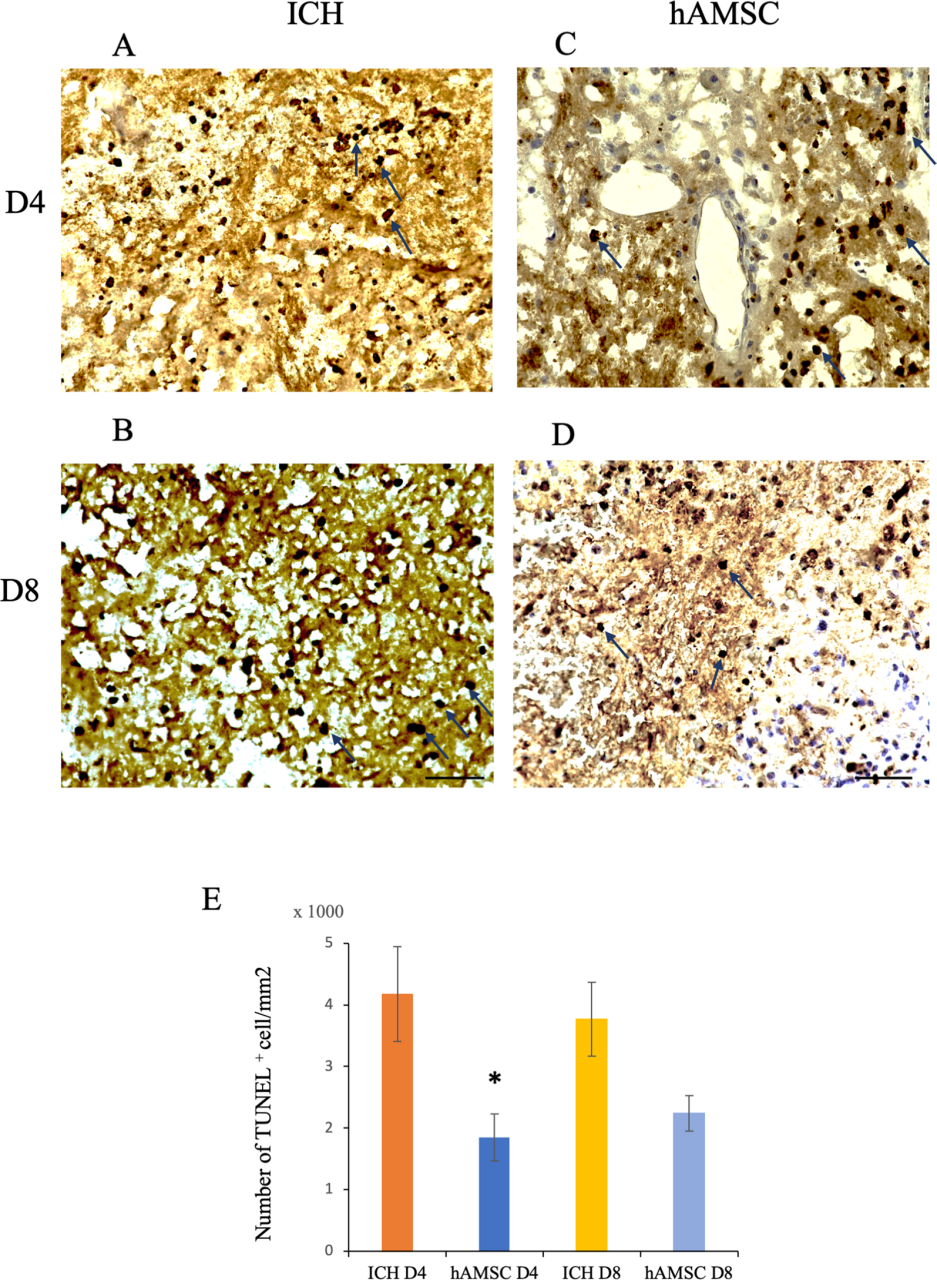
hAMSCs administration decreases the TUNEL-positive cells in the ICH lesions. Representative images of TUNEL staining of the ICH lesions at Days 4 and 8. Magnifications: ×40. **A** The ICH group at Day 4. **B** The ICH group at Day 8. **C** The hAMSC group at Day 4. **D** The hAMSC group at Day8. **E** The numbers of TUNEL-reactive cells (arrows) around the ICH lesions were enumerated. Data are plotted in mean ± SEM. *P* values are based on Wilcoxon test. *P < 0.05 compared with the same Day of ICH group. Scale bars indicate 100 μm

ICH has been reported to cause inflammation by macrophage and microglia, subsequently induce apoptosis of the brain [29, 30]. To evaluate the effects of hAMSCs on the ICH-indued apoptosis, we performed TUNEL staining and counted TUNEL-positive cells in the hAMSC and the control groups. The hAMSC groups showed TUNEL-positive cells on Days 4 at lower levels than the control group (*P* = 0.0204, Fig. 6). The hAMSC administration decreased ICH-induced apoptosis.

### Intravenous administration of hAMSCs suppresses protein expression of iNOS and TNFα

**Fig. 7:**
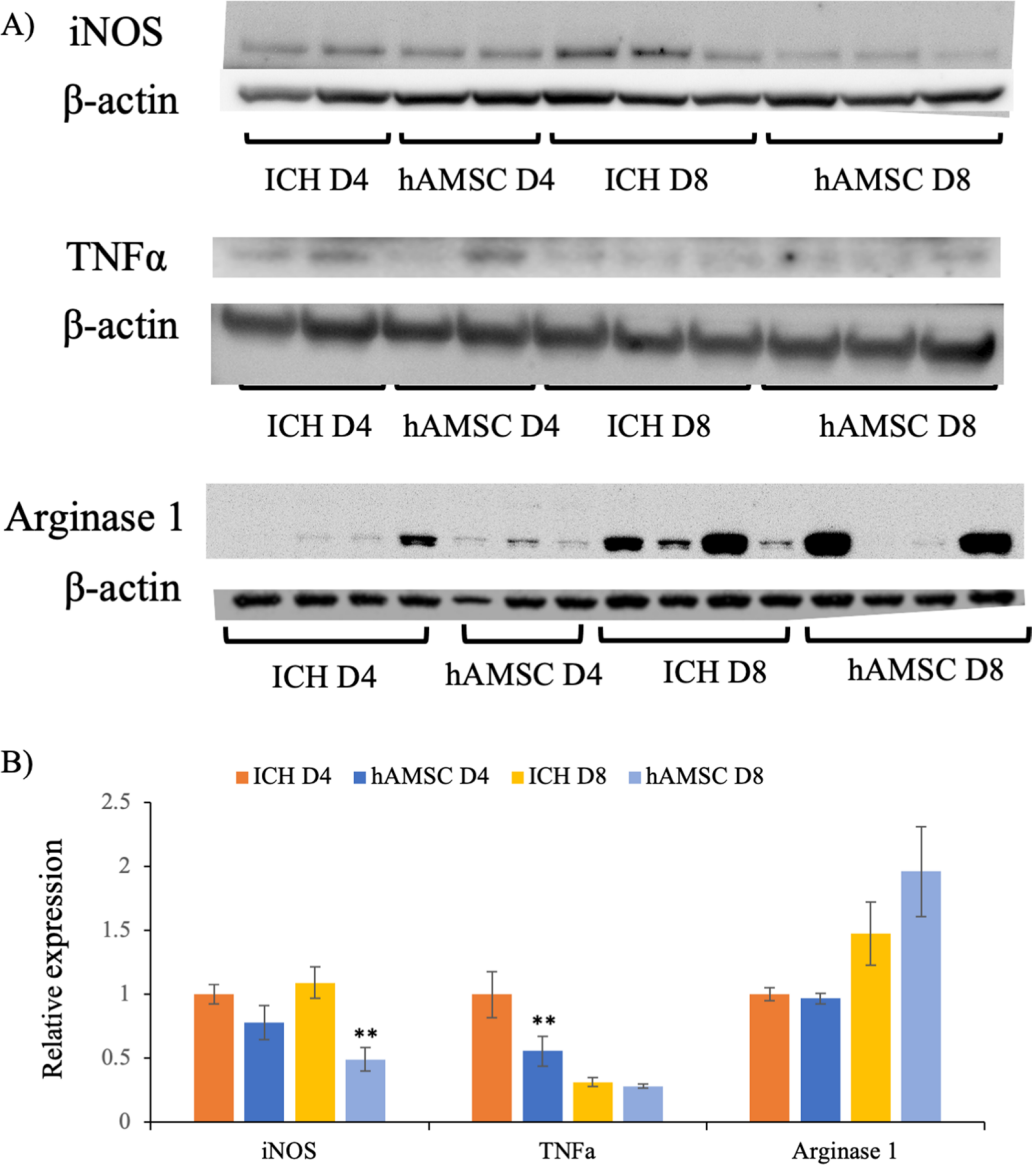
Intravenous administration of hAMSCs suppress protein expression of iNOS and TNFa. The ICH tissues were extracted and subject to Western blotting for macrophage-related factors iNOS, TNFα, and arginase 1. **A** Representative image of protein levels of iNOS, TNFα, and arginase 1. **B** Data were plotted in mean ± SEM. *P* values are based on Tukey–Kramer test. *P < 0.05 compared with the ICH group at the same day

ICH has been reported to affect macrophage-related factors (such as iNOS, TNFα, and arginase 1), transcriptional factors (such as NF-kB, STAT3, and p38MAPK), and apoptosis-related molecules (such as Caspase3) [28]. Consistent with these data, our data also demonstrated the impact of the hAMSC administration on the number of macrophages/microglia as well as apoptotic cells in the ICH lesions (Figs. 4 and 6). These findings led us to perform Western blotting to evaluate the expression levels of proteins relevant to the ICH-induced microenvironmental events. The hAMSC administration inhibited protein expression levels of TNFα at Day 4 (*P* = 0.0090) and iNOS at Day 8 (*P* = 0.00012; Fig. 7). In contrast, the hAMSC administration did not affect the expression levels of arginase 1 (Fig. 7), several transcriptional factors (p38MARK [31], NFkB [32], Akt [33], and Stat3 [34]) and apoptosis-related molecules (Caspase3 [35]; Additional file 1). These data suggest that the hAMSC administration would suppress not only the number of macrophages/microglia (Fig. 4) but their functions (iNOS and TNFα) at least partially.

### Intravenous administration of TNFa counteracts the inhibitory effect of hAMSCs on TUNEL-positive cells

**Fig. 8:**
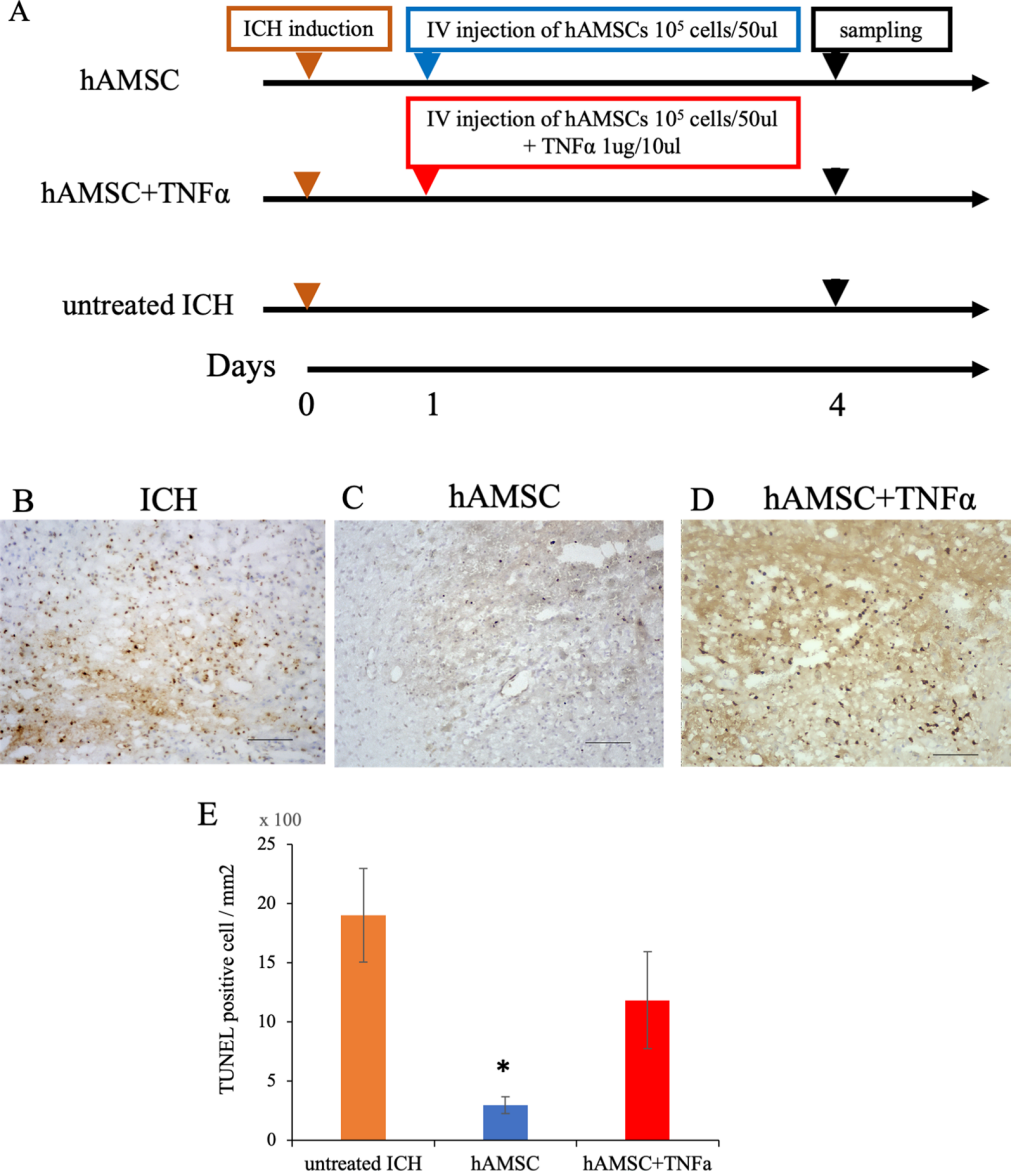
Intravenous administration of TNFα counteracts the inhibitory effect of hAMSCs on TUNEL-positive cells. **A** A protocol of TNFα rescue experiment. The ICH-bearing mice were divided into three groups: **B** the ICH group at Day 4, **C** the hAMSC group at Day 4, and **D** the hAMSC+TNFα group at Day 8. **E** The numbers of TUNEL-reactive cells around the ICH lesions were enumerated. Data are plotted in mean ± SEM. *P* values are based on Tukey–Kramer test. *P < 0.05 compared with the Day 4 of ICH group. Scale bars indicate 100 µm

To prove that hAMSCs inhibit TNFα in cell death, we administered hAMSCs and TNFα at the same time and compared them with hAMSCs alone and untreated ICH (Fig. 8A). The TUNEL-positive cells observed in the untreated ICH group significantly reduced in response to the hAMSC administration (*P* = 0.0329), which was reverted by intravenous TNFα rescue (*P* = 0.222; Fig. 8B, C). These data suggest that the inhibitory effect of hAMSCs on cell death would be mediated by macrophage-TNFα axis. Taken together, our data demonstrated that the hAMSC administration ameliorated the ICH-bearing neurobehavioral deficits more effectively compared with tenfold higher doses of hADSCs by suppressing local inflammation and apoptotic cell death in the ICH lesions.

## Discussion

This study demonstrated that the hAMSC-based treatment effectively improved the neurobehavioral deficits of the ICH-bearing mice, particularly when the treatment was initiated at Day 1 after the ICH induction. In the dose-comparison experiments (Fig. 1), the hAMSC administration promoted comparable efficacy to the hADSC administration at 1/10 to 1/40 of the cell number. In the timing-optimizing experiments (Fig. 2), initiating hAMSC treatment in the acute phase (Day 1) led to better therapeutic outcome compared with the subacute phase (Day 3). The systemically administered hAMSCs were recruited into the ICH lesions locally surrounded with host macrophages/microglia (Fig. 3). Detailed immunological evaluations revealed that the hAMSC treatment decreased the number of CD11b^+^CD45^+^ and Ly6G^+^ cells in the ICH lesions (Fig. 4), while splenocytes were not affected (Fig. 5). The hAMSC treatment decreased the number of apoptotic cells in the ICH lesions (Fig. 6). The above results were associated with suppression of the protein expression of macrophage-related factors iNOS and TNFα (Fig. 7). After the TNFα rescue, the inhibitory effect of hAMSCs on apoptosis was also reduced (Fig. 8). Taken together, these data indicate that early initiation of hAMSC treatment, even at relatively low doses, effectively ameliorates ICH-induced neurobehavioral deficits by suppressing local inflammation and apoptotic cell death in the ICH lesions.

When administered into the ICH-bearing mice, the hAMSCs demonstrated clinical efficacy equivalent to or better than that of hADSCs at 1/10 the cell number (Fig. 1). Until recently, the hAMSC dosage has not fully validated. In this regard, the present study demonstrated very powerful and impressive results that hAMSCs can achieve equivalent or better therapeutic efficacy with 1/10 of the cell number of hADSCs. Since there has been no significant difference in the culture methods of BMSCs, hADSCs, and hAMSCs used in the previous studies [11, 14, 24, 36, 37]. This finding appears attributable to the histological origin of hAMSCs. The amniotic membrane belongs to the fetus and is flexible enough to adapt to the development of the fetus and changes in the surrounding environment (such as infections). It is highly possible that physiological hAMSCs also contribute to this property of the amnion. In fact, hAMSCs have been found to have a higher capacity to produce cytokines and less rejection than other MSCs [14–16, 38]. That is to say, the hAMSCs may contribute to the suppression of fetal rejection. However, most studies have only examined cultured hAMSCs and have not elucidated the physiological functions of in situ hAMSCs in amnia. This is an interesting research topic that should be investigated in the future.

The experiments to determine the timing of hAMSC administration showed better therapeutic effects on Day 1 than on Day 3 after the ICH induction (Fig. 2), although the difference of the therapeutic effects was trivial. In this regard, since the neurological symptoms of Day 3 mice tended to recover [11], we considered them to be equivalent to ICH patients of 1–2 weeks after onset. By calculating backwards, Day 1 mice would correspond to acute ICH patients on days 1–3. In addition, considering the actual clinical situation, there may be some time lag between the ICH onset and treatment initiation. Such situation of delayed start of treatment was assumed to be the Day 3 treatment group. Regarding the timing of MSC administration, most previous studies have initiated treatment on Day 1 after disease onset as in the present study, and the earliest was 1 h and the latest was 2 months after disease onset [39]. In ICH, microglia in the brain have benn shown to be activated within an hour, and a variety of inflammatory cells infiltrate the brain within a few hours [40]. The peak of these reactions has been reported to last 3–7 days, followed by several weeks [1, 28]. Assuming that early administration of hAMSCs can decrease ICH-induced local inflammation, pre-peak administration of hAMSCs may be ideal. Similarly, our data also showed that the hADSC administration on Day 1 was more effective than on Day 3 after ICH onset. Future studies need to examine how earlier treatment (within 24 h) affects hematoma volume and brain edema.

Intravenously administered hAMSCs were found to have migrated into the brain (Fig. 3). In this regard, it remains unclear which route of administration is optimal for hAMSC treatment. There are two main routes of administration of cell therapy for intracranial lesions: intravenous and direct intracranial administration [10, 41, 42]. The advantage of intravenous administration is that it does not require any special surgical techniques or devices. This is the most realistic route when considering clinical application to humans. The disadvantage is that the administered cells are often trapped in the lungs and spleen [43], thus a larger number of cells may be required. In contrast, the advantage of direct intracranial administration is that cells can be administered near the lesion. However, the risk of local infection is unavoidable, albeit slight. In addition, and the use of stereotactic surgical systems and the development of special syringes are essential for stable and diffuse administration. Although the frequency with which intravenously administered hAMSCs reach the brain remains to be verified, the intravenous administration route appears very practical for clinical applications. Therefore, we have made it our first choice in the experimental cell therapies for CNS diseases. However, it is still unknown the ICH microenvironment milieu influences the function of hAMSCs. As cytokines greatly impact on the stemness of stem cells, the hAMSCs migrating into the ICH lesion should be exposed to various cytokines in the lesion and differentiate. In addition, it has been shown that MSCs in lesions can exist for only 3 weeks at most [44]. These findings suggest that the neuroprotective effects of the hAMSCs would occur within a very short time. In other words, hAMSCs would inhibit macrophage migration to the ICH lesions immediately after their administration and then promote the neuroprotective effects as shown in this study. As the fate and functional kinetics of hAMSCs migrating into the lesion are the most important aspect of these cells, we are currently addressing this issue as a top priority.

The hAMSC treatment suppressed the number of macrophages (and probably microglia as well) in the brain (Fig. 4), which suggests that macrophages and microglia would play important roles in chronic/subacute inflammation induced by ICH. In considering the biology of macrophages/microglia, their differentiation/polarity is of importance. Concretely, Ly6C^+^ macrophages/microglia are classified as M1 macrophages/microglia [45, 46] and are involved in the induction of acute inflammation [47, 48]. In contrast, Ly6G^+^ macrophages/microglia are classified as M2 macrophages/microglia [45, 46] and inhibit acute inflammations [49], but are also involved in chronic inflammations in asthma and COPD [50–52]. Also, the M2 macrophages/microglia have been shown to increase in the ICH lesions 3–7 days after onset [1]. M2 macrophages/microglia are currently classified into four major subtypes: M2a, M2b, M2c, and M2d [53]. M2a and M2c are typical anti-inflammatory subgroups and expressing Arginase 1 [54]. In contrast, M2b and M2d also express TNFα and iNOS [55]. M2 macrophages/microglia are generally associated with anti-inflammatory responses, but when overexpressed, they cause chronic inflammation. Which is consistent with our current and previous data (Fig. 7) [11]. However, in our model, there was no significant change in the systemic inflammatory response as shown by changes in peripheral blood and spleen (Fig. 5). Clinically, ICH is recognized as a disease that causes systemic susceptibility to infection; it is often associated with severe pneumonia and complication. There is discrepancy with the results of this study. This point needs to be re-examined in a more clinically relevant experimental system. Another issue of this study is that we did not distinguish between macrophages and microglia precisely. Macrophages and microglia in the brain are myeloid populations with distinct and shared functions, the latter of which includes phagocytosis. In this study, we only evaluated the spatial distribution of macrophage/microglial cell populations in ICH using a common marker Iba-1, which function is largely unknown. Thus, the pathological and functional differences between macrophages and microglia in the ICH remain as a major area of future research and discussion. To address this question, we are currently planning to do adoptive transfer of ex vivo-labeled macrophages into ICH-bearing mice to distinguish from microglia and evaluate the differences in their phagocytotic functions in the ICH lesions by immunohistochemistry or flow cytometry.

ICH promotes cellular damage, including cytotoxicity and oxidative stress in blood released from blood vessels as well as inflammation [56] Consistent with our data (Fig. 6), apoptosis has been observed in the ICH lesion [30]. In addition, a small number of hAMSCs directly reached the ICH lesions and inhibited apoptosis (Fig. 6). The main constituent cells of the brain are neurons and glial cells, while endothelial and pericyte cells exist around cerebral blood vessels. Based on the results of behavioral tests, it is highly likely that the hAMSC treatment at least suppress neuronal cell death. Furthermore, as MSCs can differentiate into all three germ layers, the hAMSCs themselves may contribute to neuronal regeneration in our experimental system. In this regard, we are currently working on a project on neural regeneration after stroke using BMSCs, hADSCs, and hAMSCs.

We performed Western blotting in this study (Fig. 7) to explain the underlying mechanisms at the protein levels of intracellular interaction and tissues. As a result, changes in inflammatory-related factors including TNFα and iNOS, which would contribute to the worsening of neurobehavioral symptoms, were detected in the ICH lesions followed by the hAMSC administration. In this regard, TNFα is a cytokine produced mainly by macrophages but is also produced by lymphocytes and mast cells. It is a potent mediator of inflammation and has various effects such as inducing apoptosis and immunosuppression [57]. iNOS is also produced by macrophages, CTLs, NK cells, and vascular endothelial cells; it induces inflammation through NO production [58]. However, no changes were observed in arginase-1, a representative enzyme of M2 macrophages/microglia, or in other transcriptional factors and apoptosis-related molecules, as consistent with the previous studies [59–63]. These data suggest that methods of evaluating tissues as a single mass, such as the one we used, is not suitable for evaluating ICH tissues, where changes occur in many cells. In contrast, as cell-by-cell evaluation by flow cytometry was remarkably effective in this study (Fig. 4), more detailed methods of evaluating cell-specific events such as single cell analysis would be desirable to solve these problems in the future. Another point of contention is the possibility that these related molecules may be derived from other immune cells described abobe. In our ICH mouse model, the macrophage responses would begin within a few hours after onset, and then the lymphocyte response begins on around Day 3 and later [64]. As the therapeutic window of our study was Days 1–3 after the onset of ICH, we speculated that macrophages would be the main source of these molecules during this period.

Among the molecules suggested to be involved, rescue experiments were conducted for TNFα (Fig. 8). After the TNFα rescue, the inhibitory effect of hAMSCs on apoptosis also decreased, indicating the contribution of TNFα in our ICH model. Other possible related molecules included iNOS and Arginase 1 (Fig. 7), both of which are enzymes involved in macrophage/microglia functions. Because they are enzymes, they were not suitable for systemic administration in vivo and must be manipulated specifically in macrophages in the brain. This point remains to be verified in future studies.

## Conclusion

In conclusion, the present study demonstrated that intravenous hAMSC administration during the acute phase would efficiently improve ICH-induced neurobehavioral disorders. The underlying mechanism was suggested to be the suppression of subacute inflammation and apoptosis by suppressing macrophage/microglia cell numbers and macrophage/microglia functions (such as TNFα and iNOS). From a clinical point of view, hAMSC-based treatment may be a novel strategy for the treatment of ICH.

## Supplementary Information

**Additional file 1.** Intravenous administration of hAMSCs does not affect transcriptional and apoptosis-related molecules. Western blotting in the brain ICH targeting according to transcriptional and apoptotic-related molecules. **A** Representative images of protein levels. **B** Data are plotted in mean ± SEM. *P* values are based on Tukey–Kramer test. *P < 0.05 compared with the ICH group at the same day.

## Data Availability

The datasets used and/or analyzed during the current study are available from the corresponding author on reasonable request.

## Abbreviations

ICH: Intracerebral hemorrhage
hAMSC: Human amnion-derived stem cell
hADSC: Human adipose-derived stem cell
BMSC: Bone marrow-derived stem cell
MSC: Mesenchymal stem cell
iNOS: Inducible nitric oxide synthase
TNFα: Tumor-necrosis factor alpha
