# Supplementary figures and images for: Early-phase administration of human amnion-derived stem cells ameliorates neurobehavioral deficits of intracerebral hemorrhage by suppressing local inflammation and apoptosis
Source: J Neuroinflammation. 2022 Feb 12;19:48. doi: 10.1186/s12974-022-02411-3 (PMC8840774; doi:10.1186/s12974-022-02411-3)

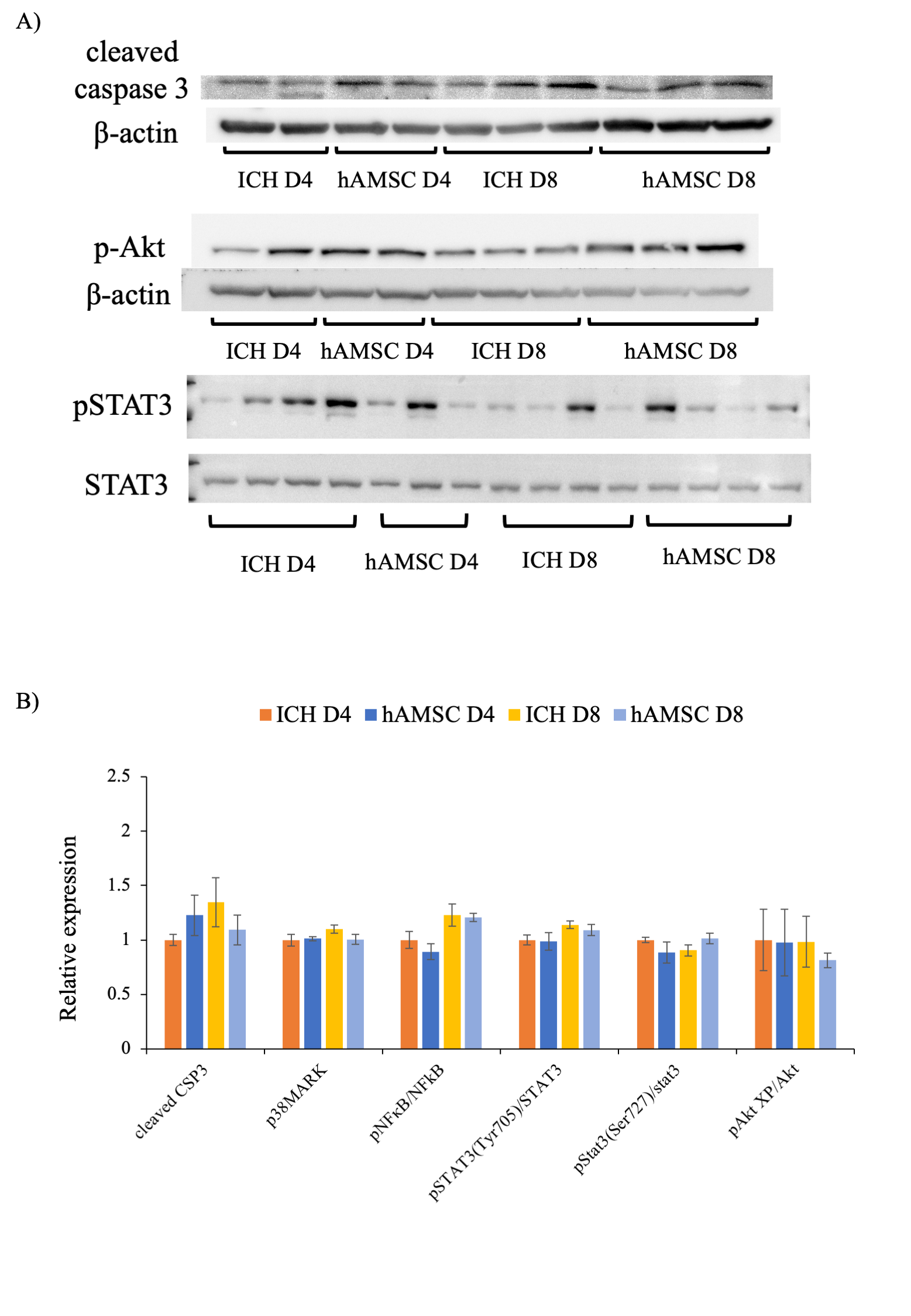

Supplement: Supplementary file 1 — Additional file 1. Intravenous administration of hAMSCs does not affect transcriptional and apoptosis-related molecules. Western blotting in the brain ICH targeting according to transcriptional and apoptotic-related molecules. A Representative images of protein levels. B Data are plotted in mean ± SEM. P values are based on Tukey–Kramer test. *P < 0.05 compared with the ICH group at the same day. [file 12974_2022_2411_MOESM1_ESM.tiff]
